# ﻿An unexpected new red-bellied *Stumpffia* (Microhylidae) from forest fragments in central Madagascar highlights remaining cryptic diversity

**DOI:** 10.3897/zookeys.1104.82396

**Published:** 2022-06-06

**Authors:** Katherine E. Mullin, Manoa G. Rakotomanga, Jeff Dawson, Frank Glaw, Andolalao Rakotoarison, Pablo Orozco-terWengel, Mark D. Scherz

**Affiliations:** 1 Cardiff University, School of Biosciences, Sir Martin Evans Building, Museum Avenue, Cardiff, CF10 3AX, UK Cardiff University Cardiff United Kingdom; 2 Conservation Action Plan for Madagascar (‘C.A.P. Mada’), Antananarivo, Madagascar Conservation Action Plan for Madagascar (‘C.A.P. Mada’) Antananarivo Madagascar; 3 Durrell Wildlife Conservation Trust, Les Augrès Manor, La Profonde Rue, Trinity, Jersey, JE3 5BP, Channel Islands, UK Durrell Wildlife Conservation Trust, Les Augrès Manor Jersey United Kingdom; 4 Zoologische Staatssammlung München (ZSM-SNSB), Münchhausenstr. 21, 81247 München, Germany Zoologische Staatssammlung München (ZSM-SNSB) Munich Germany; 5 Department of Animal Biology, University of Antananarivo, Madagascar University of Antananarivo Antananarivo Madagascar; 6 School for International Training, VN 41A Bis Ambohitsoa Ankazolava, 101 Antananarivo, Madagascar School for International Training Antananarivo Madagascar; 7 Natural History Museum of Denmark, University of Copenhagen, Universitetsparken 15, 2100, Copenhagen Ø, Denmark University of Copenhagen Copenhagen Denmark

**Keywords:** Amphibian, cophyline, DNA barcoding, phylogeny, taxonomy

## Abstract

The Madagascan endemic subfamily Cophylinae in the family Microhylidae, is an example of a taxonomic group for which much is still to be discovered. Indeed, the cophyline frogs present a large portion of Madagascar’s cryptic and microendemic amphibian diversity, yet they remain understudied. A new red-bellied species of the microhylid frog genus *Stumpffia* is described from the central plateau of Madagascar. Visual encounter surveys in Ambohitantely and Anjozorobe in 2019 and 2020 identified this previously unknown *Stumpffia* species, which closely resembles *Stumpffiakibomena* known from Andasibe in the east. *Stumpffialynnae***sp. nov.** adds another species to the red-bellied species complex, differing from *S.kibomena* by genetic differentiation in the mitochondrial 16S rRNA gene (3.6–3.9%) and distinct nuclear RAG1 haplotypes, as well as strongly by its advertisement call. The new species is known from across Ambohitantely Special Reserve and Anjozorobe Angavo protected area, but is known only from one complete specimen and eight individual tissue samples. Based on the rarity of the species, the small number of locations in which it has been found, and its disappearing forest habitat, its IUCN Red List classification is suggested as “Endangered”. This species is the first *Stumpffia* described from Madagascar’s central plateau, highlighting the importance of conserving the remnant forest fragments in this area and the ongoing need to survey and protect this threatened habitat type.

## ﻿Introduction

Madagascar is one of the world’s top biodiversity hotspots for conservation priority (Myers et al. 2000) and is home to a predicted 500+ species of endemic amphibians (Perl et al. 2014), although only 375 have been described so far (Frost 2022). Despite intensive studies in the last 30 years, there are still significant gaps in the taxonomic inventory of anurans (Vieites et al. 2009). Meanwhile, much of the island’s forest is now reduced to remnant forest fragments with data from 2014 showing that 46% of Madagascar’s forest is within 100 m of a forest edge (Vieilledent et al. 2018). The loss and fragmentation of this key amphibian habitat puts its endemic species at risk of extinction, making full inventories necessary to understand the island’s extant biodiversity, and to guide conservation efforts of these environments.

The three Madagascan endemic subfamilies of Microhylidae Günther, 1858; Dyscophinae Boulenger, 1882, Scaphiophryninae Laurent, 1946, and Cophylinae Cope, 1889; are examples of taxonomic groups for which much is still to be discovered. Indeed, the cophyline frogs present a large proportion of Madagascar’s cryptic and microendemic amphibian diversity (Rakotoarison et al. 2017); however they are understudied. The subfamily Cophylinae is a morphologically diverse group comprising arboreal, terrestrial, fossorial, and rupicolous species (Glaw and Vences 2007). Within this subfamily, *Stumpffia* Boettger, 1881 are the most diverse of the eight recognized genera, with currently 44 recognised species (Frost 2022) and several species still to be described (Rakotoarison et al. 2017).

*Stumpffia* contains most of the smallest cophyline frogs (Rakotoarison et al. 2017), some of which are among the smallest vertebrates in the world, with adults generally having a snout–vent length (SVL) under 20 mm and some species reaching only 9 mm SVL (Glaw and Vences 2007; Klages et al. 2013; Rakotoarison et al. 2017). The genus is found across much of the humid parts of the island, but most species are found in northern Madagascar. Several species of *Stumpffia* are micro-endemic (Wollenberg et al. 2008; Köhler et al. 2010) with many species restricted to very small ranges, sometimes restricted to single mountaintops (Rakotoarison et al. 2017).

Prior to surveys in 2018 no *Stumpffia* species were known from Madagascar’s central plateau (Ambohitantely Special Reserve and surrounding forest fragments). *Anilanyhelenae* Vallan, 2000 from Ambohitantely Special Reserve was originally described as *Stumpffiahelenae* (Vallan, 2000a), before a comprehensive revision of the Cophylinae determined this species to be in its own genus (Scherz et al. 2016), leaving the central plateau again without any *Stumpffia*.

Visual encounter surveys in late 2018 (Razafindraibe et al. 2021), early 2019, and 2020, however, discovered a *Stumpffia* species of uncertain affinities that had a bright red belly. According to general morphological similarities to *Stumpffiakibomena*Glaw et al. 2015, this species was called Stumpffiacf.kibomena by Razafindraibe et al. (2021). During the same surveys in 2020 an apparently similar *Stumpffia* was also recorded 70 km east of Ambohitantely in the Anjozorobe-Angavo protected area, an area containing more continuous forest than Ambohitantely, and which was until relatively recently connected to Madagascar’s eastern rainforest block. The only other *Stumpffia* record for this site was *Stumpffiaroseifemoralis* Guibé, 1973 which was found in a 2007 inventory (Wilme et al. 2007) and was reported as scarce, found in just one of seven surveyed locations. In a more recent review inventory, this one *Stumpffia* species record was listed as *Stumpffia* sp., not *S.roseifemoralis* (Goodman et al. 2018). The identification of this species originally as *S.roseifemoralis* was probably based on the red colouration of its legs, but its identity as *S.roseifemoralis* can be ruled out because this species has been shown to be restricted to north-eastern Madagascar (Rakotoarison et al. 2017) and is morphologically different to the individuals found in the 2020 surveys.

Bright red colour on the venter is an interesting feature that has recently been highlighted as occurring in several different clades of frogs in Madagascar (Glaw et al. 2020). In *Stumpffia*, several species are known to have red or reddish colouration over their posterior abdomen and ventral surfaces of legs: *Stumpffiabe*Köhler et al., 2010, *S.kibomena*, *S.meikeae*Rakotoarison et al., 2017 , *S.miovaova*Rakotoarison et al., 2017, *S.nigrorubra*Rakotoarison et al., 2017 and *S.roseifemoralis* (Rakotoarison et al. 2017). These species belong to several different clades, and such colouration must have evolved independently in this genus, and assignment to any one species, or even clade, solely based on the red colour is not possible.

Geographically, the nearest occurring species of *Stumpffia* with a red belly is *S.kibomena*, described by Glaw et al. (2015) from the Andasibe region in central-eastern Madagascar. It is known from two areas in the Andasibe vicinity, but is rarely seen and few specimens are available, leading to the assumption that this species has a secretive lifestyle, is seasonal, or is indeed rare and restricted to a small range. *Stumpffiakibomena* belongs to a diverse clade of frogs of moderately large body size (14.4–23.7 mm), called Clade C2 by Rakotoarison et al. (2017), which includes several lineages with red bellies (*S.miovaova* and *S.kibomena*, and several deep lineages considered conspecific with *S.kibomena* by Rakotoarison et al., (2017)), but others with no remarkable ventral colour (*Stumpffiaachillei*Rakotoarison et al., 2017, *S.analanjirofo*Rakotoarison et al., 2017, *S.fusca*Rakotoarison et al., 2017) and one species with stark white and black ventral colouration (*S.grandis* Guibé, 1973). These species are all distributed in rainforests of eastern and northeastern Madagascar.

Here, we provide new data on the red-bellied *Stumpffia* species that has been found in both Ambohitantely and Anjozorobe. We find that it is morphologically and genetically most similar to *Stumpffiakibomena*, but that it differs substantially from that species genetically and especially in the male advertisement call. We therefore describe it as a new species, which represents the first species of the genus in the central highlands of Madagascar.

## ﻿Materials and methods

### ﻿Specimen collection and morphological measurement

**Figure 1.**
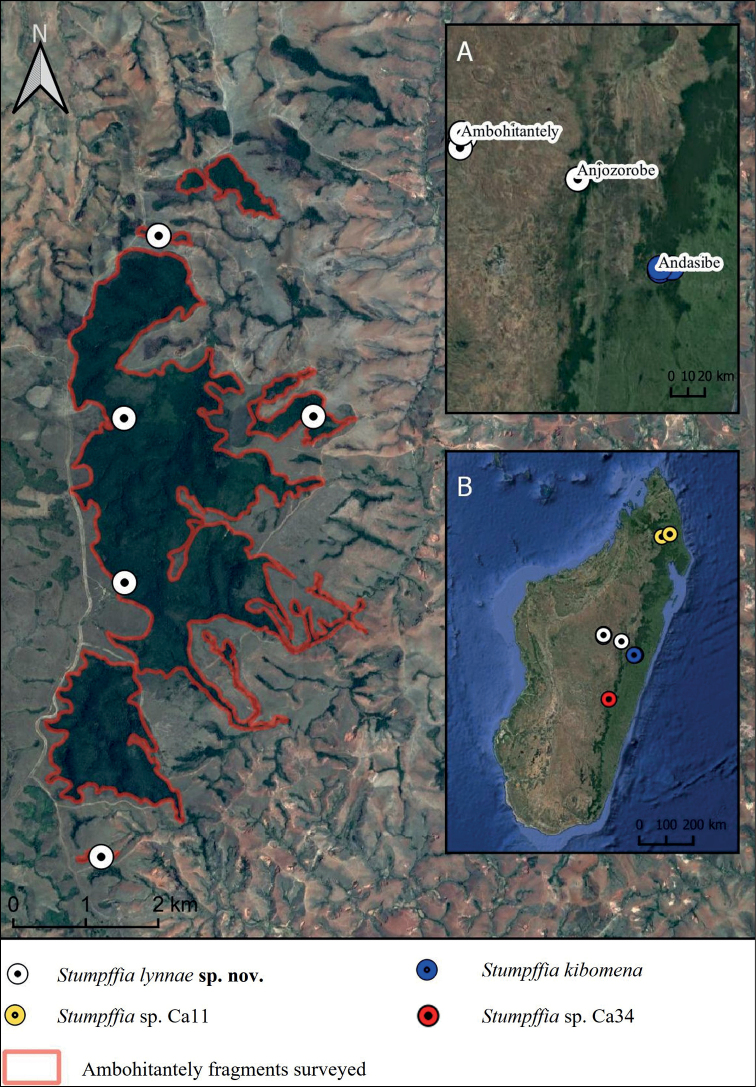
Map of Ambohitantely Special Reserve showing known *Stumpffialynnae* sp. nov. distribution. **A** Ambohitantely, Anjozorobe, and Andasibe relative to one another **B** map of Madagascar showing the wider distribution of *Stumpffialynnae* sp. nov. and its sister species *Stumpffiakibomena*, *Stumpffia* sp. Ca11 and *Stumpffia* sp. Ca34. Satellite imagery Google Earth (2015).

Visual encounter surveys (**VES**) were conducted during the day and evening throughout March – May 2019 and January – March 2020 in some of the last remaining forest blocks in Madagascar’s central highlands. Locations included six forest fragments in Ambohitantely Special Reserve (18.1960°S, 47.2865°E, elevation ~ 1600 m a.s.l.), the two forest fragments in Ankafobe protected area (18.1089°S, 47.1932°E, elevation 1475 m a.s.l.), forest at Anjozorobe (18.4095°S, 47.9447°E, elevation ~ 1350 m a.s.l.), and at Andasibe Mitsinjo (18.9335°S, 48.4129°E, elevation ~ 900 m a.s.l.) (Fig. 1). VES were conducted along 200 m transects to allow sampling across the different microhabitats and biotypes present in the forest fragments, and quadrat samplings (4 m^2^) were also conducted randomly along the transects to target leaf litter species (Bell et al. 2006). Our survey methods followed the ‘intermediate intensity’ of VES, returning all objects to their original position and not destroying any habitat features, reducing impact on the environment (Crump and Scott 1994).

DNA was sampled from each individual using the less invasive buccal swab sampling technique, following the method of Pidancier et al. (2003). Fine tip rayon swabs (Medical Wire & Equipment Co. #MW113) were used, and stored in 0.5–0.75 mL of Longmire Lysis buffer (Longmire et al. 1997). When individuals were too small to buccal swab, skin swabs were taken instead. Field numbers used were **KAMU**, **KAMUS**, referring to the collections of Katherine Mullin, specimen and swabs respectively. Institutional acronyms are as follows: **ZFMK**, Zoologische Forschungsmuseum Alexander Koenig, Bonn; **NMBE**,Naturhistorisches Museum Bern; and **ZSM**, Zoologische Staatssammlung München. The snout–vent length (SVL) measurement was taken of all individuals in situ, and all individuals were photographed in life. One specimen from Ambohitantely (field number KAMU2) was collected on 21 January 2020. It was euthanised using Benzocaine, fixed in 100% ethanol, and stored in 70% ethanol for long-term preservation. Tissue was removed from the right thigh and stored in 100% ethanol. The specimen was deposited in the ZSM. The following measurements of the preserved specimen were taken by KM using precision callipers, as in Rakotoarison et al. (2017): snout–vent length (**SVL**), maximum head width (**HW**), head length (**HL**), horizontal tympanum diameter (**TD**), horizontal eye diameter (**ED**), eye-nostril distance (**END**), nostril–snout tip distance (**NSD**), nostril–nostril distance (**NND**), forelimb length (**FORL**), hand length (**HAL**), hindlimb length (**HIL**), foot length including tarsus (**FOTL**), foot length (**FOL**), tibia length (**TIBL**), and tibio-tarsal articulation (**RHL**).

### ﻿Molecular datasets

DNA was extracted from the buccal swabs and storage lysis buffer using the Qiagen DNeasy kits following manufacturer’s protocol. We assembled phylogenies based on two mitochondrial gene fragments to assess the phylogenetic relationship between the newly collected material and other *Stumpffia* species, and built a haplotype network using one nuclear gene.

The 5’ fragment of 16S rRNA (~ 630 base pairs) was amplified using the 16SL3 and 16SAH primers as in Vences et al. (2003). A 12.5 µl PCR reaction volume was used including 2 µl of DNA (concentration not quantified), 1 µl of 5 × Green GoTaq Flexi reaction buffer (Promega), 1.5 µl of MgCl_2_ (25 mM), 0.6 µl of deoxynucleotides (dNTPs 10mM/each), 0.3 µl of each primer (10pmol), 0.06 µl of 5U/μl GoTaq G2 Flexi DNA Polymerase (Promega), and molecular biology-grade deionised H_2_O. PCR thermo-cycling conditions were as follows: denaturation for 90 s at 94 °C, followed by 33 cycles of denaturation at 94 °C for 45 s, primer annealing at 55 °C for 45 s and PCR product extension at 72 °C for 90 s, finishing with an elongation step of 72 °C for 10 min. This region is not the usual 16S rRNA region used for barcoding frogs in Madagascar, but has previously been used to assess mitochondrial differentiation in *Stumpffia* (Rakotoarison et al. 2017), enabling comparisons to be made. PCR products were sequenced at Eurofins Genomics and the sequences viewed and edited in Geneious Prime. Published sequences of 27 other *Stumpffia* species were downloaded from GenBank and aligned with our new sequences using the MUSCLE alignment algorithm in MEGAX (Kumar et al. 2018). These species included representatives from all major clades of *Stumpffia* (12 in Clade A, one in Clade B, four in Clade C1, and ten in Clade C2). Model testing was also conducted in MEGAX and the most suitable DNA evolution model was selected with the AIC criterion. A Maximum Likelihood phylogeny was constructed, also in MEGAX, using the GTR+G model, using all sites, and Subtree-Pruning and Regrafting (SPR) Level 5 that carries out an extensive search of the tree space. Uncorrected pairwise distances (p-distance) were calculated between the species used in the phylogeny using the TaxI2 tool in the iTaxoTools toolkit (Vences et al. 2021), which is based on the original TaxI (Steinke et al. 2005).

The recombination-activating gene 1 (**RAG1**) was used as a nuclear marker as it is known to show distinct haplotypes for closely related *Stumpffia* species (Klages et al. 2013; Rakotoarison et al. 2017). These sequences were analysed separately from the mitochondrial DNA in order to obtain further support of their status as a distinct species, as it provides evidence for genetic differentiation of lineages from an unlinked locus (Vences et al. 2018). The primers CophF1 and CophF2 were used to amplify a region of RAG1 (~ 503 bp), using the same reaction volume and concentrations as above, and with PCR thermo-cycling conditions of 120 s of denaturation at 94 °C, followed by 35 cycles of denaturation at 94 °C for 20 s, primer annealing at 53 °C for 50 s and PCR product extension at 72 °C for 180 s, finishing with a final PCR product elongation step of 72 °C for 10 min, as in Rakotoarison et al. (2019). PCR products were sequenced in both directions to enable the reliable identification of heterozygote sites. Sequences were checked and heterozygote positions inferred in Geneious Prime. The novel sequences were aligned with those of closely related species in Clade C2 of Rakotoarison et al. (2017) monograph using MUSCLE, ensuring all sequences were on the same reading frame. Sequences were not available for *Stumpffia* sp. Ca11 and *S.betampona*Rakotoarison et al. 2017. The final alignment, and that used for the haplotype network, was 338 bp in length due to the length of the sequences available for comparison.

Haplotypes were phased in DNAsp (Rozas et al. 2017) using the PHASE algorithm (Stephens, et al. 2001). A Neighbour-Joining tree based on uncorrected pairwise differences was constructed in MEGAX and visualised as a haplotype network using Haploviewer (http://www.cibiv.at/~greg/haploviewer).

A fragment of mitochondrial Cytochrome Oxidase 1 (COI) was also amplified and sequenced, however given there is limited reference material from closely related species (1 sequence from *Stumpffia* sp. Ca34 and none from *Stumpffia* sp. Ca11 or *S.kibomena*), it was not analysed in detail here. The primers Chmf4 and Chmr4 were used to amplify a region of ~ 658 bp, using the same reaction volume and concentrations as above, and with PCR thermo-cycling conditions as in Che et al. (2012) with the exception of a higher annealing temperature at 58 °C. To add support of the placement of this putative new species in the *Stumpffia* phylogeny a Maximum Likelihood phylogeny was constructed using the HKY+G+I model with 1000 bootstrap replicates. The same *Stumpffia* species as those in the 16S tree were used, but fewer species have COI sequences available, and hence the dataset is smaller.

Where possible the sequences of the same individual within each species were used throughout the 16S, RAG1 and COI analysis for consistency. All newly obtained sequences were deposited into GenBank (accession numbers are provided in Suppl. material 3: Table S1). To contribute toward the growing reference barcoding database for Malagasy amphibians, we also amplified the 3’ fragment of 16S rRNA using the 16SA-L and 16SB-H primers (Palumbi et al. 1991).

### ﻿Bioacoustic analysis

One advertisement call recording was made of a male observed with an inflated vocal sac in Anjozorobe. The call recording was made using the application RecForge II on a Samsung Galaxy A5 smartphone using its internal microphone, the file was saved as a .wav sound file. This male was swabbed, and DNA sequenced. Air temperature at the time of the call was recorded with a Kestrel 2500 weather meter. Call analysis was conducted in Audacity v. 2.3.3, following a call-centred approach, defining a call as the main coherent sound unit, separated from other such units by a distinct period of silence, with multiple calls strung together into a call series (Köhler et al. 2017). Temporal call parameters are given in milliseconds (**ms**) and the number of calls analysed (***n***) in parentheses. Recordings were re-sampled at 44.1 kHz and 32-bit resolution for analysis. The call file contained at least two individuals calling, but only the target specimen (the loudest calls) was included in our analysis. We silenced the inter-call intervals between the eight calls, amplified the calls by 10 decibels, and carried out one round of noise reduction. Spectrograms were obtained with a Hanning window function at 1024 bands FFT resolution. The frequency analysis tool in Audacity was used to check for the most dominant energy peak, and this was repeated for each call in the call series. This analysis was also conducted with a Hanning window function at 1024 bands. We also measured the *Stumpffiakibomena* calls from Vences et al. (2006) (CD 3, track 52) to ensure the calls were measured in the same way. The original call measurements from the *S.kibomena* species description (Glaw et al. 2015) were also collated for comparison.

## ﻿Results

Throughout 880-person survey hours during both day and evening surveys, only eight individuals of the putative new *Stumpffia* species were observed, six from Ambohitantely and two from Anjozorobe (Table 1). Morphologically they strongly resemble *S.kibomena* and two candidate species; *Stumpffia* sp. Ca11 from Marojejy and Ambolokopatrika in north-eastern Madagascar, and *Stumpffia* sp. Ca34 from Ranomafana National Park.

**Table 1.** Details of all *Stumpffialynnae* sp. nov. samples from Ambohitantely (Amb) and Anjozorobe (Anj). ND: Not Determined.

Sample ID	Date	Location	Time	Coordinates	Elevation (m)	Forest type	Distance from water (m)	Substrate	Sex	SVL (mm)	Call	COI	5’ 16S rRNA	RAG1
KAMUS60	13/04/19	Amb	10:15	S18.1969, E47.2842	1586	Slope	10	Leaf litter	Juvenile	8.3	–	X	X	X
KAMUS74	16/04/19	Amb	19:29	S18.1753, E47.3088	1408	Riparian	1	Leaf litter	ND	15.5	–	X	X	–
KAMUS167	12/05/19	Amb	11:10	S18.2328, E47.2811	1522	Slope	5	Soil under large rock	ND	19.8	–	X	X	X
KAMUS200	16/05/19	Amb	08:50	S18.1517, E47.2886	1532	Slope	5	Leaf litter	ND	11.3	–	X	X	X
ZSM 1/2022 holotype	24/01/20	Amb	09:00	S18.1517, E47.2886	1540	Riparian	5	Leaf litter under pandans	ND	20.9	–	X	X	X
KAMUS256	24/01/20	Amb	09:53	S18.1517, E47.2886	1528	Riparian	5	Leaf litter	ND	16.0	–	X	X	X
KAMUS370	15/02/20	Anj	19:00	S18.4116, E47.9501	1432	Slope	ND	Leaf litter	F – gravid	22.2	–	X	X	X
KAMUS371	15/02/20	Anj	19:00	S18.4116, E47.9501	1432	Slope	ND	Leaf litter	M – calling	20.1	X	X	X	X
Razafindraibe et al. (2021)	12/19	Amb	08:30-11:00	S18.1755, E47.2841	1560	ND	ND	Bamboo node	ND	~15-20	–	–	–	–

### ﻿Molecular species delimitation

**Figure 2.**
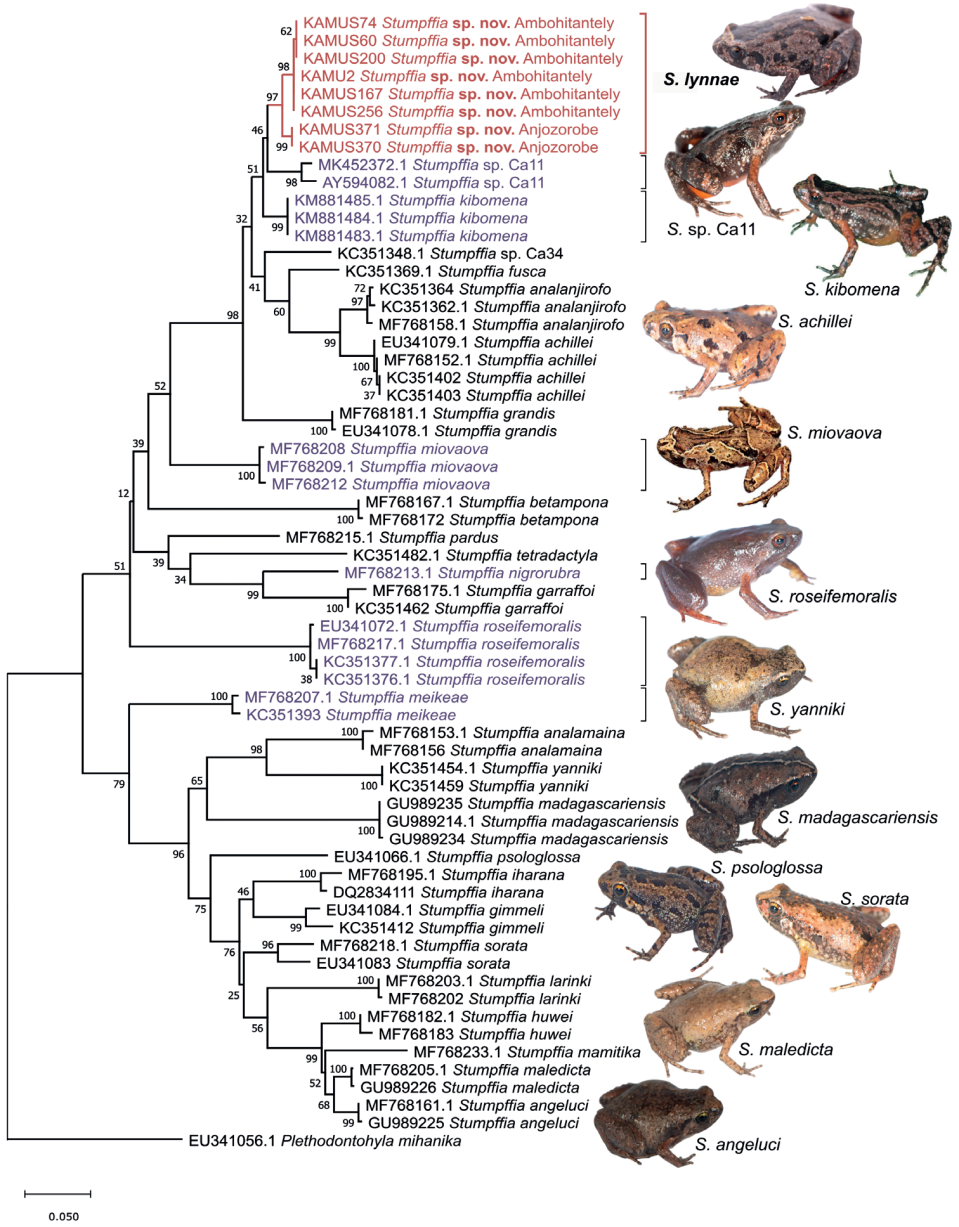
Maximum Likelihood tree of *Stumpffia* spp. based on analysis of a 621 bp fragment of the mitochondrial 16S rRNA gene. MUSCLE alignment, General Time Reversible model plus Gamma distribution, all sites used, × 1000 bootstraps, SPR level 5, no variant sites. Purple labels mark the other red-bellied *Stumpffia* species. Photographs Mark D. Scherz and Frank Glaw.

Maximum Likelihood analysis of the 5’ fragment of the mitochondrial 16S rRNA gene of the species included in the analysis yielded a phylogeny which reflected that seen in the comprehensive integrative taxonomy conducted by Rakotoarison et al. (2017) (Fig. 2). The new specimens from Anjozorobe and Ambohitantely formed a well-supported clade (97% bootstrap; Fig. 2), divided into two subclades by locality also well supported statistically (98% Ambohitantely clade and 99% Anjozorobe clade). As predicted based on their morphology, the putative new species seems to share a common ancestor with *Stumpffia* sp. Ca11 and *S.kibomena*, although support for the clade including these three species was low (51%).

Uncorrected pairwise distances (p-distances) estimated for the 16S sequences between specimens from Anjozorobe and Ambohitantely was 1.3%, while the within groups distances were 0.0% and 0.12%, respectively. The mean p-distance between the Ambohitantely individuals and the *S.kibomena* sequences was 3.9%, and between the Anjozorobe individuals and *S.kibomena* sequences was 3.6% (Suppl. material 4: Table S2). This exceeds the threshold of 3% used to consider a candidate species in Neotropical and Malagasy frogs (Fouquet et al. 2007; Vieites et al. 2009). Similar levels of divergence were found between *Stumpffia* sp. Ca11 and the putative new species; *Stumpffia* sp. Ca11 is 4.2% divergent with respect to the Anjozorobe samples, 4.6% with respect to the Ambohitantely sequences, and 4.5% from *S.kibomena*. Meanwhile *Stumpffia* sp. Ca34 had larger p-distances, being 7.0–7.2% divergent from the putative new species (no difference between Ambohitantely and Anjozorobe individuals), 6.0% from *S.kibomena*, and 7.2–8.0% from *Stumpffia* sp. Ca11. There are currently a lack of specimens and knowledge available for the descriptions of *Stumpffia* sp. Ca11 and *Stumpffia* sp. Ca34 (Rakotoarison et al. 2019).

Within the 16S alignment there are five differences that are unique to the putative new species (Suppl. material 2: Fig. S2). These include (1) one position where all eight specimens have a C while all other *Stumpffia* in the alignment have a T (position 480 in the alignment), and (2) another position where all eight specimens have a T and all other included species have a C with the exception of *S.betampona* which has an A (position 545). The other three differences vary between the specimens from Ambohitantely and those from Anjozorobe; (3) one position, which is a T in the six Ambohitantely specimens but is a C in all other species and the Anjozorobe specimens (position 141), (4) a two base pair deletion in the two individuals from Anjozorobe (CC or CT depending on the species they are compared to; positions 196–197), and (5) the insertion of an A in the six Ambohitantely individuals that is not present in any other species or the specimens of Anjozorobe (position 456).

**Figure 3.**
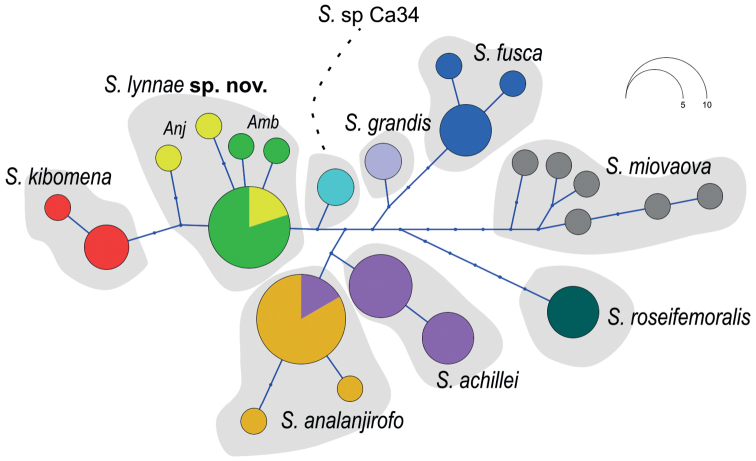
Haplotype network constructed from nuclear RAG-1 gene sequences (338 bp) of seven specimens of *Stumpffialynnae* sp. nov. (two from Anjozorobe (Anj; yellow) and five from Ambohitantely (Amb; green)) and eight other closely related *Stumpffia* species from Clade C2 (Rakotoarison et al. 2017). Small dots represent mutational steps.

The RAG-1 sequence (504 bp) was successfully obtained for seven out of the eight individuals from Ambohitantely and Anjozorobe. The sequences obtained show segregating genetic variation (five haplotypes) and one haplotype shared between Ambohitantely and Anjozorobe (Fig. 3). Neither of these localities shared haplotypes with individuals of *S.kibomena* or the other species, and they differed from them by at least three mutational steps.

The COI phylogeny mirrors that of the 16S marker, and also forms a well-supported clade of the putative new species (100% bootstrap), with the Anjozorobe individuals divided into a well-supported subclade (99%). The putative new species is sister to *Stumpffia* sp. Ca34 (81% support). The phylogeny can be seen in Suppl. material 1: Fig. S1.

In summary, there is concordant evidence from two mitochondrial markers and a nuclear DNA fragment to support the species-level genetic distinction of the specimens from Anjozorobe and Ambohitantely, not just from Clade C2 species but also from all 27 described species of *Stumpffia* included in the analysis, as well as from the nearest currently known candidate species, *Stumpffia* sp. Ca11 and *Stumpffia* sp. Ca34.

### ﻿Acoustic differentiation

**Figure 4.**
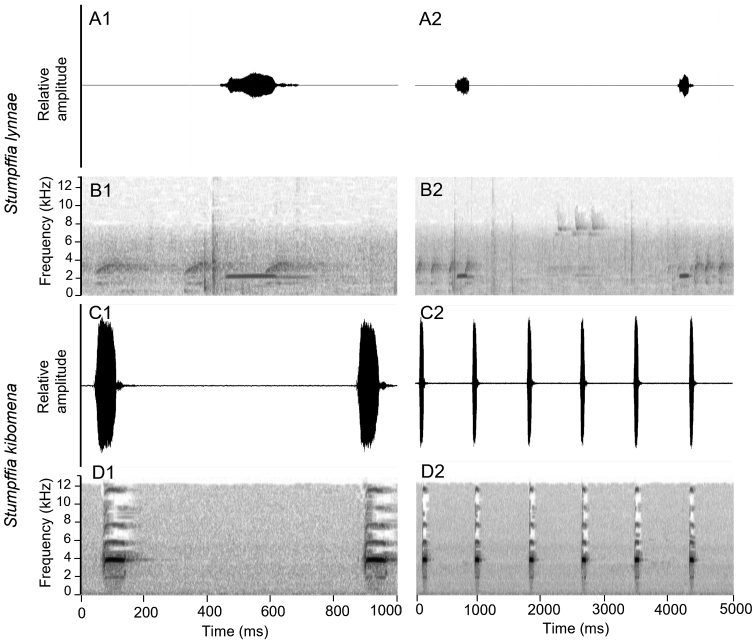
Male advertisement call of *S.lynnae* sp. nov. **A** oscillogram **B** spectrogram. *S.kibomena***C** oscillogram **D** spectrogram over a one-second interval (column 1) and a five-second time interval (column 2).

The advertisement call of *S.kibomena* was described by Glaw et al. (2015). Here, we reanalysed an available call recording of that species and the calls of one specimen of the putative new species from Anjozorobe (Fig. 4, Table 2; no call voucher available). Distinct differences are recognizable between these calls. Note/call duration and dominant frequency are suitable variables for taxonomic inference (Köhler et al. 2017) and both of these call traits are drastically different from those of calls of *S.kibomena*, as is the duration of intervals between calls in the call series (Fig. 4, Table 2). Call duration (= note duration) was longer in the putative new species than in *S.kibomena* (163–184 ms vs. 68–82 ms (*n* = 8) based on our measurements of the *S.kibomena* calls), and the duration of intervals between calls was also much longer (3498–5581 ms vs. 742–766 ms in *S.kibomena* (*n* = 7)). The dominant frequency range was lower (2027–2044 Hz vs. 3858–3883 Hz in *S.kibomena*). These differences are such that the two calls can easily be distinguished by human ear, providing unambiguous support for the species-level distinction of these two taxa. Regrettably, no calls are available from *Stumpffia* sp. Ca11 or *Stumpffia* sp. Ca34.

**Table 2.** Acoustic traits of *Stumpffiakibomena* in comparison to those of *S.lynnae* sp. nov.

Species and source	Number of calls in series	Call – repetition rate (number calls/second)	Call duration (= note duration) (ms)	Duration of interval between calls (end-start) (ms)	Dominant frequency range (Hz)
*S.kibomena* (Glaw et al. 2015)	11–22 (*n* = 5)	1.2–1.3	70–76 (73±2, *n* = 9)	770–813 (797±15, *n* = 9)	3900–4300
*S.kibomena* (CD track measured by KM)	21	1.2	68–82 (74.3, *n* = 8)	742–766 (757, *n* = 7)	3858–3883
*Stumpffialynnae* sp. nov. (KM original recording)	8	0.25	163–184 (174, *n* = 8)	3498–5581 (4357, *n* = 7)	2027–2044

Thus, in the following we provide a formal description of this new species, which is morphologically cryptic with respect to *S.kibomena* but genetically and bioacoustically distinct.

#### ﻿ Stumpffia lynnae sp. nov.

Taxon classificationAnimaliaAnuraMicrohylidae

68237AD8-8E7D-5E68-B421-EE9061AD2D84

http://zoobank.org/C5DD7133-3EC6-46D9-A895-48864C56EB61

##### Holotype.

**Figure 5.**
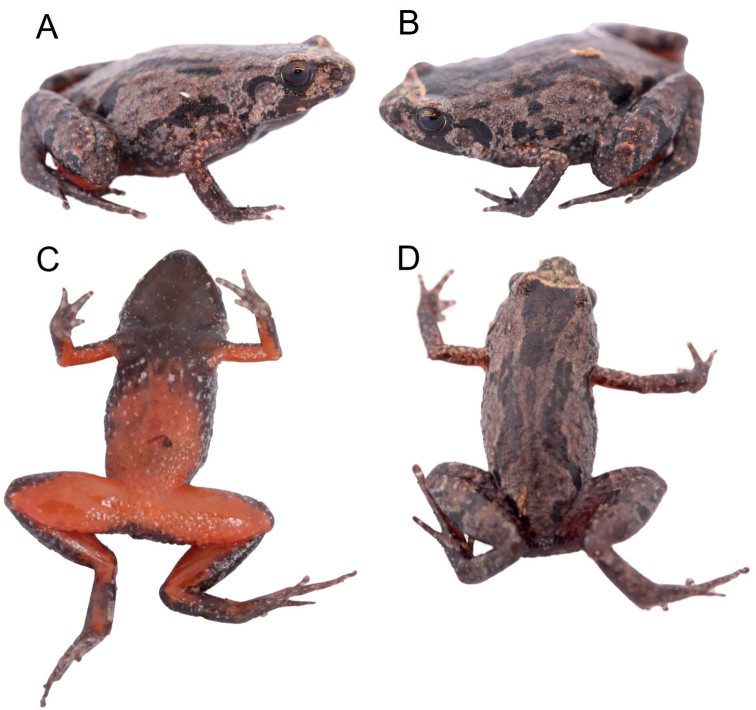
Images of the holotype ZSM 1/2022 (KAMU2) from Ambohitantely Special Reserve in life **A, B** dorsal lateral view **C** ventral view **D** dorsal view.

ZSM 1/2022 (field number KAMU2), an unsexed adult, collected by K. Mullin, M. G. Rakotomanga, M. L. C. Razafiarimanana and T. Raditra, on 21 January 2020, in one of the northern fragments in Ambohitantely Special Reserve (18.1517°S, 47.2886°E, 1540 m a.s.l.), Analamanga Region, central Madagascar (Fig. 5).

##### Diagnosis.

The new species is assigned to the genus *Stumpffia* based on its morphological and genetic affinities. Within the genus, it is distinguished by the unique combination of the following characters: (1) SVL 15.5–22.2 mm (adults in life), (2) limited digital reduction on the hands and feet such that first finger is reduced; other fingers not reduced and first toe is slightly reduced; other toes not reduced, (3) bright red to orange colouration confined to the ventral surfaces of the legs, posterior abdomen, and ventral arms, (4) absence of red markings on the lower jaw, and advertisement call with (5) inter-call intervals of 3498–5581 ms, (6) call/note duration 163–184 ms, (7) dominant frequency 2027–2044 Hz, and (8) distinct genetic divergence in the mitochondrial and nuclear genome to other known species.

*Stumpffialynnae* sp. nov. can be distinguished from all other *Stumpffia* species except *S.be*, *S.kibomena*, *S.meikeae*, *S.miovaova*, *S.nigrorubra*, and *S.roseifemoralis* by the presence of bright red colouration on ventral surfaces of arms, legs, and abdomen. Among these species it can be distinguished from *S.be* by smaller body size (15.5–22.2 mm adults in life vs. 25.2 mm) and less expanded terminal discs on fingers and toes, as well as being in a different major clade of *Stumpffia* (Clade C rather than Clade A; Rakotoarison et al. 2017); from *S.meikeae* by the colour on the belly (bright red to orange vs. fainter more champagne to salmon colouration in *S.meikeae*), by less expanded terminal discs on fingers and toes, as well as being in a different major clade of *Stumpffia* (Clade C rather than Clade B; Rakotoarison et al. 2017); from *S.miovaova* by a larger maximum body size (22.2 vs. 18.2 mm) and colour on the belly (bright red vs. fiery orange); from *S.nigrorubra* by the dorsal colouration and patterns (dark brown dorsolateral bands and pale brown colouration in adult *S.lynnae* vs. darker mottled black and dark iridescent dorsal colouration in *S.miovaova*, and its phylogenetic placement in Clade C2 rather than Clade C1 (Rakotoarison et al. 2017); and finally from *S.roseifemoralis* by a larger maximum body size (22.2 vs. 18.4 mm), more vibrant ventral colouration, rougher dorsal skin in life, and less homogeneous dorsal colouration. Further, *S.lynnae* sp. nov. differs from all the above, other than *S.kibomena*, by the presence of dense blackish pigmentation on the throat, and different dorsal patterns.

**Figure 6.**
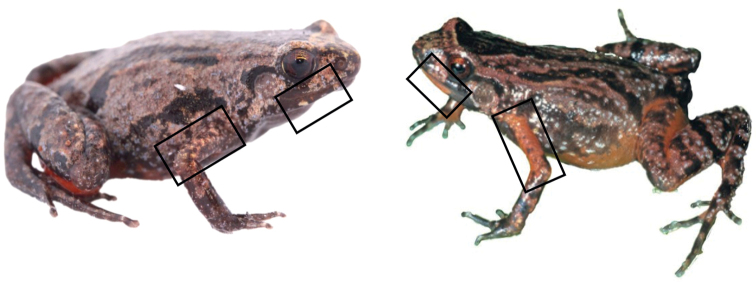
Direct comparison between the holotype of *S.lynnae*ZSM 1/2022 (KAMU2) and a female *S.kibomena* specimen from An’ala (photo Frank Glaw). Boxes highlight the morphological differences highlighted; elongated red marking on each side of the lower jaw on *S.kibomena*, absent on the observed *S.lynnae*, and the red arms on *S.kibomena* compared to the brown arms on *S.lynnae*.

**Figure 7.**
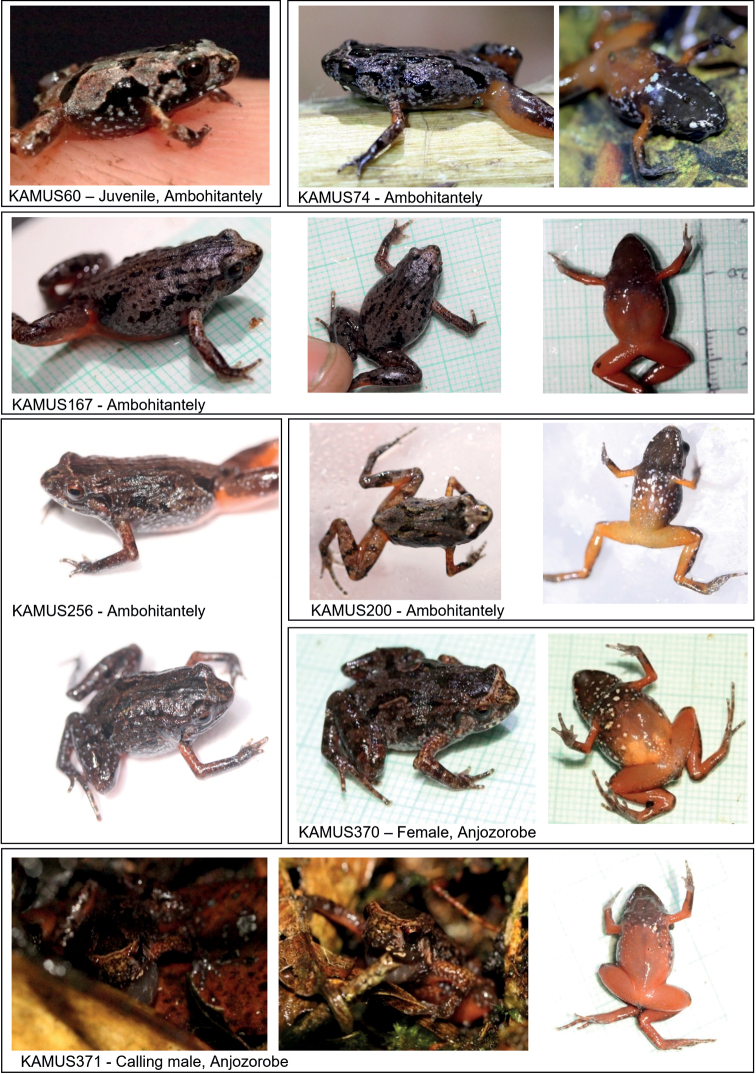
Images of the additional observed specimens of *Stumpffialynnae* sp. nov. including the calling male (KAMUS371).

**Figure 8.**
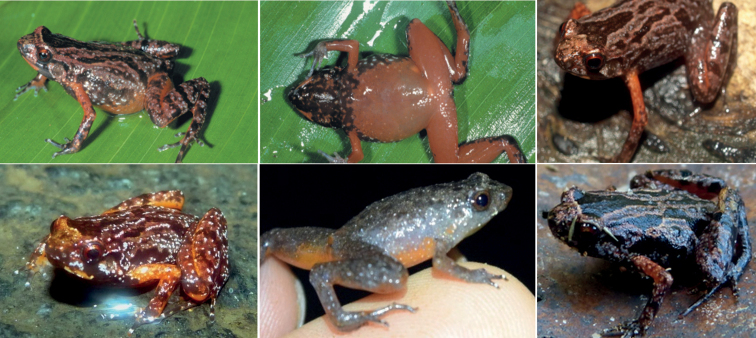
Images of *Stumpffiakibomena* (top row from the species description of Glaw et al. (2015) and the bottom row from inaturalist.org taken by Leonard Bolte and Jordan Broadhead, used with permission). Note the red marking on the lower lips and the red arms which differ from *S.lynnae* sp. nov.

*Stumpffialynnae* sp. nov. is morphologically almost indistinguishable from *S.kibomena* (Table 3). However, an elongated red marking on each side of the lower jaw is absent from all observed specimens, but can be present in *S.kibomena* (Glaw et al. 2015), and the upper arms of *S.lynnae* are usually brown versus usually red in *S.kibomena*. Direct comparison of these features can be seen in Fig. 6, with additional ‘in life’ images of the *S.lynnae* individuals sampled and *S.kibomena* specimens in Figs 7, 8, respectively, for further comparison. The new species also strongly differs from *S.kibomena* in bioacoustics (see Table 2 and the comparison of the two species in the previous section). It differs specifically from *S.kibomena* by the following base pair differences in the analysed 5’ fragment of the 16S rRNA gene: position 385 T vs. C and 547 A vs. T. It further differs from *S.kibomena* and all other *Stumpffia* species in our alignment by the base pair differences as listed above: position 480 C vs. T; 545 T vs. C or A; 141 T (Ambohitantely specimens) vs. C; 196-197 CC/CT deletion (Anjozorobe specimens); and 456 A insertion (Ambohitantely specimens).

**Table 3.** Morphological data of *Stumpffialynnae* sp. nov. and *Stumpffiakibomena* (data for latter species from Glaw et al. (2015), and the proportion of the SVL (/SVL).

Measurements (mm)		*S.lynnae* sp. nov. Holotype ZSM 1/2022	/SVL	*S.kibomena* holotype ZFMK 60007	/SVL	*S.kibomena*NMBE 1044940	/SVL	*S.kibomena*NMBE 1034211	/SVL
1	SVL	20.5		21.2		19.8		17.1	
2	HW	6.5	**0.32**	6.7	**0.32**	6.9	**0.35**	6.2	**0.36**
3	HL	5.0	**0.24**	5.1	**0.24**	4.9	**0.25**	4.3	**0.25**
4	TD	1.5	**0.07**	1.2	**0.06**	1.4	**0.07**	1.3	**0.08**
5	ED	2.0	**0.10**	1.9	**0.09**	1.6	**0.08**	1.6	**0.09**
6	END	1.5	**0.07**	1.9	**0.09**	1.7	**0.09**	1.3	**0.08**
7	NSD	0.5	**0.02**	1.1	**0.05**	0.8	**0.04**	0.8	**0.05**
8	NND	2.0	**0.10**	2.4	**0.11**	2.4	**0.12**	2.3	**0.13**
9	FORL	12.0	**0.59**	12.5	**0.59**	12.1	**0.61**	11.5	**0.67**
10	HAL	4.0	**0.20**	5.0	**0.24**	4.5	**0.23**	4.4	**0.26**
11	HIL	32.0	**1.56**	35.5	**1.67**	32	**1.62**	29.5	**1.73**
12	FOTL	14.5	**0.71**	15.3	**0.72**	14.7	**0.74**	14.8	**0.87**
13	FOL	8.5	**0.41**	10.0	**0.47**	8.9	**0.45**	9.2	**0.54**
14	TIBL	9.0	**0.44**	10.6	**0.50**	9.6	**0.48**	9.3	**0.54**
15	RHL	Eye		Eye		Eye		Eye	

##### Holotype description.

**Figure 9.**
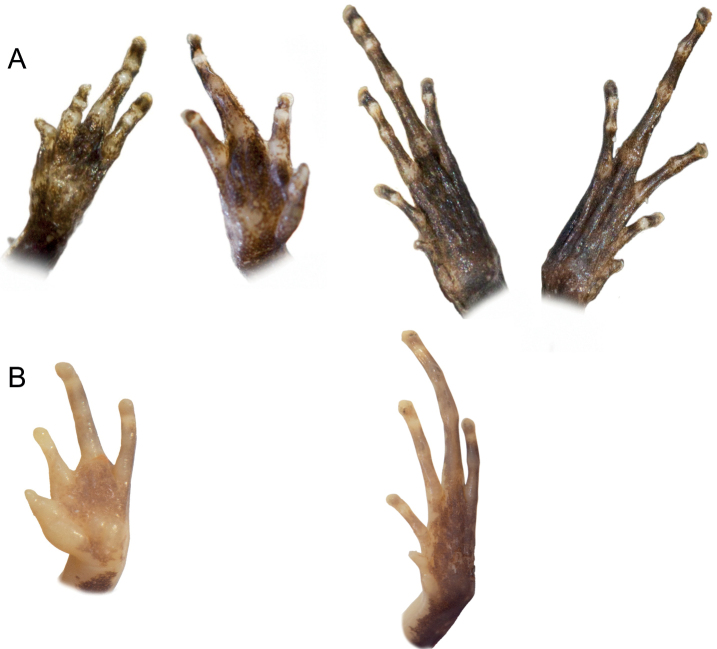
Holotype hands and feet of *S.lynnae* (row **A**) and *S.kibomena* (row **B**). Not to scale.

A specimen in a good state of preservation, except for skin loss where left thigh tissue muscle was removed for tissue sample. The body is elongated with the head wider than long, but not wider than the body. The snout is roughly rounded in both dorsal and lateral views. The nostrils are not protuberant and are closer to the tip of the snout than the eye. The tympanum is distinct and large (75% of eye diameter), and the supratympanic fold is indistinct. First finger short, others not reduced (Fig. 9). Inner metacarpal tubercle low, without a distinct prepollex, outer metacarpal tubercle indistinct and pale. Forelimbs are slender, the hand is without webbing, presents a relative finger length 1<2<4<3 with the fourth finger slightly longer than the second; and fingertips are not expanded into discs. First finger reduced; other fingers not reduced. The hind limbs are slender and the tibiotarsal articulation reaches the eye when the hind limb is adpressed along the body. There is no webbing between toes, and the relative toe length is 1<2<5<3<4 with the fifth toe slightly shorter than the third. First toe slightly reduced; other toes not reduced. Inner metatarsal tubercle oblong and indistinct, and outer metatarsal tubercle absent, lateral metatarsalia connected. The dorsal skin was slightly bumpy in life, without distinct dorsolateral folds, and the ventral skin was granular on the abdomen but smooth on the throat. The tongue is long, broadening posteriorly, attached anteriorly, not notched. Maxillary teeth and vomerine odontophores are absent, and the choanae are large and oval shaped.

Measurements (in mm). Snout-vent length 20.5, maximum head width 6.5, head length from tip of snout to posterior edge of snout opening 5.0, horizontal tympanum diameter 1.5, horizontal eye diameter 2.0, distance between anterior edge of eye and nostril 1.5, distance between nostril and tip of snout 0.5, internarial distance 2.0, forelimb length (from limb insertion to tip of longest finger) 12.0, hand length, to the tip of the longest finger 4.0, hind limb length (from the cloaca to the tip of the longest toe) 32.0, tibia length 9.0, foot length including tarsus 14.5, foot length 8.5 (Table 3).

In life the dorsum was pale brown, with dark brown markings (Fig. 5). These markings include a dark brown triangular marking between the eyes which extends posteriorly. A beige coloured band runs between the eyes at the base of the triangular marking. As in the *S.kibomena* holotype, two continuous narrow black bands extend posteriorly from level of the arms’ insertion, converging towards the mid-dorsum and then diverging posteriorly without touching towards the inguinal region (referred herein as dorsolateral bands). The throat is black with few scattered white spots and these white spots continue as the ventral side becomes bright red. The pupil is black with a copper iris and golden colouration at the top. All four limbs are coloured red on the ventral sides. After two years in preservative, the red ventral colouration has faded.

##### Variation.

Seven additional frogs were sampled for DNA and basic measurements were taken. Details of these frogs and their collection sites are shown in Table 1 and images in Fig. 7. There is some variation in colour/patterns with some individuals being greyer on the dorsal side (rather than brown) (KAMUS60 and KAMUS74), also with dark brown markings. The markings of the juvenile are black. The dorsolateral bands are not always present, for example KAMUS167 has speckled markings. This individual also did not have the beige band across the head between the eyes. The female from Anjozorobe (KAMUS370), KAMUS256 and KAMUS200 from Ambohitantely had pale brown markings outlining the dark brown dorsolateral bands. All specimens have the same ventral colouration and markings (black throat, red belly, and white spots), though the tone of the belly varies from orange, dark orange to red (KAMUS167; see Fig. 7).

##### Call description.

The calls were recorded from a male (KAMUS371) at Anjozorobe during heavy rainfall on the 15^th^ of February 2020, at 19:30, at an air temperature of 20.5 °C. The male was calling while sitting on the leaf litter on a slope in primary forest close to a female. The male was not collected as a specimen due to lack of permits to allow this, but swabs for DNA analysis were collected. The advertisement call of *S.lynnae* sp. nov. is structurally similar to that of other *Stumpffia* species in that it is a single melodious note that is repeated at regular intervals in call series. The call is simple, with the call composed of a slightly amplitude-modulated single tonal note, with multiple calls repeated in series at regular intervals. The calls are evenly spaced across the call series with silent intervals between calls. A definitive number of calls per call series cannot be determined given the sample size of one recording. Frequency is distributed across one band for each note, and frequency modulation is relatively equal across the note. No harmonics were seen in this call recording. The individual emitted eight calls, with a call-repetition rate of 0.25 calls per second. Call/note duration was 163–184 ms, and the duration of intervals between calls was 3498–5581 ms (*n* = 7). The dominant frequency range was 2027–2044 Hz (Table 2).

##### Etymology.

This species name is a matronym honouring Lynne Mullin, to whom we are pleased to dedicate this attractively colourful species in recognition of the unconditional support she has provided to the first author. The origin of Lynn/e is from Celtic language, with the meaning waterfall, pond, and lake. Given the popular waterfall in the centre of Ambohitantely Special Reserve where this species was first found, this name seems appropriate. The name has further relevance to this beautiful red-bellied frog with the Spanish meaning of the feminine name ‘pretty’. The species epithet is defined as a genitive noun with the ‘e’ removed for easier pronunciation.

##### Distribution.

While just eight individuals were recorded, the six at Ambohitantely were distributed across four fragments (three in addition to the core forest block) including a very small (3.5 ha) fragment at the southern end of the reserve (Fig. 1). This suggests that they are widely distributed across the protected area. Surveys in the two forest fragments at Ankafobe did not detect the species, but this is not surprising given the size of the fragments and the reduced diversity at Ankafobe compared to Ambohitantely (Mullin et al. 2021). However, it cannot be ruled out that this species may exist in relict forest fragments in the area surrounding Ambohitantely Special Reserve, and between Ambohitantely and Anjozorobe. This species’ elevational range (1432–1586 m) is greater than *S.kibomena*’s range at Andasibe (900–950 m).

##### Natural history.

The encounters of the nine frogs (including the one individual found by Razafindraibe et al. 2021) were during both morning and evening surveys from December to May, suggesting they are active throughout the day and the wet season. No surveys have been undertaken outside of this wet season window, so their activity during the dry season is unknown. The holotype was resting on top of the leaf litter under a *Pandanus* sp. screwpalm in riparian habitat at an elevation of 1540 m a.s.l. All eight frogs from this study were found in slope or riparian forest, between 1 and 10 metres from a water source. All individuals were found on the forest floor; seven were on leaf litter and one was found on bare soil under a large rock. The individual recorded by Razafindraibe et al. (2021) was found guarding eggs in a water filled bamboo node. This was the first record of a *Stumpffia* laying eggs in a bamboo hole but supports evidence that closely related *Stumpffia* may reproduce opportunistically in water-filled cavities close to the forest floor (Razafindraibe et al. 2021). The advertisement call was recorded during heavy rain in the evening during February. Combined with the eggs observed in December, this suggests that this species may be reproductively active throughout the wet season. Given the low number of individuals found across the high number of survey hours, we conclude that this species either has cryptic habitats, or is indeed rare.

##### Conservation.

This species is known to occur in two locations with different conservation situations. Ambohitantely Special Reserve, currently managed by Madagascar National Parks is highly fragmented, and is threatened by cattle grazing, illegal logging and forest activities, forest burning for charcoal, and forest fires and fire suppression activities (Goodman, Raherilalao and Wohlauser 2018 pp 1338–1340). The number of forest fragments surrounding the Reserve boundary has substantially declined since 1996 (Vallan 2000b), as has the forest cover inside the reserve, having decline 6.3% from 1996–2016 (Goodman et al. 2018). Reforestation efforts within the Reserve have been blighted by fires (KM pers. obs.). Further, an invasive caterpillar is currently causing canopy leaf loss across the Reserve (S. Goodman pers. comms) which has been noted to dry out the leaf litter which could be devastating for amphibians (KM pers. obs). Meanwhile, Anjozorobe is still connected to large expanses of continuous forest, and is managed by the local-led Association Fanamby; however, it is subject to similar threats and lost 33.2% of its forest between 1996 and 2016 (Goodman et al. 2018: 1362–1365). Stricter conservation actions and management are required at both sites. We can assume that the populations of *S.lynnae* are declining due to the ongoing habitat loss and severe fragmentation of the populations. Given the very low numbers of individuals found during surveys we assume this species is naturally rare.

Under the IUCN Red List criterion B (Geographic range), we believe this species should be listed as Endangered for both B1, extent of occurrence (EOO), and B2, area of occupancy (AOO) (IUCN 2012). Its EOO is < 5,000 km^2^ at 530 km^2^; however, much of this area is inhospitable savannah grasslands and farmland between Ambohitantely and Anjozorobe. More relevant is its AOO, which is < 500 km^2^, estimated to be ~ 100 km^2^. The species fulfils two further criteria to be considered Endangered. B(a) ‘severely fragmented OR number of locations’ as it exists in severely fragmented locations/populations with no connectivity between the fragments at Ambohitantely, or between Ambohitantely and Anjozorobe. It was found in two locations across five forest fragments, one of which was just 3.5 hectares in size. Given that all the forest fragments it was found in are frequently burned and logged, they are predicted to reduce in size, with some disappearing in the near future. This degradation will influence all of the subcriteria within B(b) ‘continuing decline observed, estimated, inferred or projected in any of; (i) extent of occurrence; (ii) area of occupancy; (iii) area, extent and/or quality of habitat; (iv) number of locations or subpopulations; and (v) number of mature individuals.’ This suggested Red List status is in line with two microendemic amphibian species that are found at Ambohitantely, *Anilanyhelenae* and *Anodonthylavallani*, which are both listed as Critically Endangered given they are found in just Ambohitantely Special Reserve (*A.vallani*) (IUCN 2020a) and Ambohitantely and surrounding isolated forest fragments (*A.helenae*) (IUCN 2016, 2020b; Mullin et al. 2021).

## ﻿Discussion

*Stumpffialynnae* is genetically and bioacoustically different to its close relative *S.kibomena*, to which it is morphologically very similar. This new species of *Stumpffia* brings the total number of known *Stumpffia* to 45, but given the similarity to *S.kibomena*, *Stumpffia* sp. Ca11, and *Stumpffia* sp. Ca34, also highlights the cryptic diversity that exists within the genus.

Razafindraibe et al. (2021) and this study were the first to report this species from Ambohitantely despite surveys by Vallan (2000b) and regular surveys by field courses led by the Association Vahatra. This highlights the cryptic diversity that remains to be found in Ambohitantely and the surrounding forest fragments in the central highlands, and the importance of this area in Madagascar for amphibian diversity. It affirms the importance of continuing to survey these areas to understand the diversity present, and the addition of another potentially Endangered species supports the need to protect these areas from ongoing deforestation and forest exploitation. This delayed finding following previous surveys suggests that this species is rare and/or cryptic and elusive.

Madagascar presents high levels of amphibian micro-endemism (Wollenberg et al. 2008; Vences et al. 2009, 2010; Brown et al. 2016) and this is notable in the genus *Stumpffia* (Rakotoarison et al. 2017). There is no comprehensive hypothesis explaining why this occurs on the island across taxa, but the patterns have been explored on the basis of river catchments, bioregions, and previous climatic events causing forest contraction (Wilme et al. 2006). In terms of anurans, the correlation of body size and micro-endemism has been explored (Wollenberg et al. 2011), as has the influence of the bioclimatic zones (Brown et al. 2016). However, the relationship is still unclear given that many range to body size relationships are understudied with a lack of records and range maps. Ongoing surveys of understudied areas, such as those that facilitated our findings, will further help add to species range maps and contribute towards understanding micro-endemism, and whether for some species their small range is the result of habitat loss or understudied locations, as opposed to true micro-endemism.

The presence of *S.lynnae* in both Ambohitantely and Anjozorobe may provide evidence that these two forests were once connected. If these forests were once connected and the species existed across a larger range, this species may not have formerly exhibited micro-endemism. Its current AOO fits within the range of most *Stumpffia*, between 50 to 100 km^2^ (Rakotoarison et al. 2017), however if forest previously existed between the two sites, its range would have been much larger. When *Stumpffia* are known from more than one locality, they often present distinct mitochondrial haplotypes between the locations (Rakotoarison et al. 2017), as seen in *S.lynnae*. This pattern may be due to their small body size and limited dispersal ability, which contributes to facilitating lineage sorting (Orozco-Terwengel et al. 2011; Pabijan et al. 2012; Crottini et al. 2019). However, this genetic differentiation may also be due to forest isolation if it has persisted for a long time.

A study of endemism in the Mantellidae family found that many sister species pairs did not have overlapping ranges, but there were some examples of young microendemic sister species occurring in full sympatry (Wollenberg et al. 2011). The former reflects the relationship between *S.lynnae* and *Stumpffia* sp. Ca11, which may be more closely related than *S.lynnae* and *S.kibomena*, yet they are hundreds of kilometres apart, with *Stumpffia* sp. Ca11 distantly in the north of the island and probably having never had overlapping ranges. Future studies need to verify the status of this candidate species, as well as the poorly known *Stumpffia* sp. Ca34 from Ranomena.

On the contrary, however, the latter finding of Wollenberg et al. (2011) could mirror the case of *S.lynnae* sp. nov. and *S.kibomena*, which may have evolved in sympatry or parapatry, given the likely recent connectivity between Anjozorobe and Andasibe. Satellite imagery from the year 2000 shows much more forest between Anjozorobe and Mantadia National Park, close to Andasibe (Global Forest Watch 2021), and the forest cover shown in 1953 was greater still (Vieilledent et al. 2018). If these two species (*S.lynnae* and *S.kibomena*) did at some point occur in sympatry, this may explain the extreme difference in their male advertisement calls, which are the basis for sexual selection and mate recognition, and consequently often differ substantially between closely related sympatric taxa (Köhler et al. 2017). Meanwhile, elevation may have been a cause of allopatric divergence with respect to *S.kibomena* given that the species exists at ~ 900 m a.s.l (Glaw et al. 2015) while *S.lynnae* was found at higher elevation of ~ 1400–1600 m a.s.l. Conversely, the species may not have had overlapping ranges and the different calls may have simply been selected in different directions in isolation. More research is required to understand fully when these forests, and that at Ambohitantely, were separated, e.g., using population genetic approaches and genomic data (e.g., ddRAD-seq) from multiple species distributed in this region.

This species adds another to the list of those with red markings, which occur in many different groups of frogs in Madagascar (Glaw et al. 2020). The function of such markings is unknown and may indeed vary by clade. For example, in the mantellid frogs like *Mantellamadagascariensis*, it serves as aposematism, but in other species it may serve in antipredator behaviour or intraspecific communication (Glaw et al. 2020). In the Cophylinae, where it occurs in species of *Platypelis*, a few *Rhombophryne* species, and several independent clades of *Stumpffia*, the function is not known (Glaw et al. 2020). In this new species and in other *Stumpffia* the colour is rapidly lost when stored in 70% ethanol, whereas it is not lost in *Platypelisranjomena*Glaw et al., 2020 even after years in ethanol. This difference suggests that distinct chemical basis may underpin what otherwise may seem to be the same or similar phenotypes, as seen for *Mantellamadagascariensis* Grandidier, 1872 species group (Crottini et al. 2019). Future research could be conducted to understand the origins of this colouration.

This new *Stumpffia* highlights the importance of continued survey effort in the central highlands, and adds another endemic species to the area, highlighting the need to protect the rapidly dwindling forest fragments. It also highlights the taxonomic research that is still required to fully understand the *Stumpffia* genus, with this species alone still requiring more knowledge. More individuals should be surveyed to understand more about their population size, distribution, and population trends as well as their life history and ecology.

## Supplementary Material

XML Treatment for Stumpffia lynnae

## Supplementary materials

Supplementary material 1 Figure S1 Data type docx file Explanation note Maximum Likelihood phylogeny of selected *Stumpffia* species using a 624 bp region of the COI mitochondrial marker, using the HKY+G+I model with 1000 bootstrap replicates. https://binary.pensoft.net/file/691973This dataset is made available under the Open Database License (http://opendatacommons.org/licenses/odbl/1.0/). The Open Database License (ODbL) is a license agreement intended to allow users to freely share, modify, and use this Dataset while maintaining this same freedom for others, provided that the original source and author(s) are credited.Katherine E. Mullin, Manoa G. Rakotomanga, Jeff Dawson, Frank Glaw, Andolalao Rakotoarison, Pablo Orozco-terWengel, Mark D. Scherz

Supplementary material 2 Figure S2 Data type docx file Explanation note Sections of the 16S rRNA gene alignment, showing the differences of *Stumpffialynnae* sp. nov. in comparison to 27 other *Stumpffia* species from across the *Stumpffia* phylogeny. https://binary.pensoft.net/file/691974This dataset is made available under the Open Database License (http://opendatacommons.org/licenses/odbl/1.0/). The Open Database License (ODbL) is a license agreement intended to allow users to freely share, modify, and use this Dataset while maintaining this same freedom for others, provided that the original source and author(s) are credited.Katherine E. Mullin, Manoa G. Rakotomanga, Jeff Dawson, Frank Glaw, Andolalao Rakotoarison, Pablo Orozco-terWengel, Mark D. Scherz

Supplementary material 3 Table S1 Data type docx file Explanation note GenBank accession numbers. https://binary.pensoft.net/file/691975This dataset is made available under the Open Database License (http://opendatacommons.org/licenses/odbl/1.0/). The Open Database License (ODbL) is a license agreement intended to allow users to freely share, modify, and use this Dataset while maintaining this same freedom for others, provided that the original source and author(s) are credited.Katherine E. Mullin, Manoa G. Rakotomanga, Jeff Dawson, Frank Glaw, Andolalao Rakotoarison, Pablo Orozco-terWengel, Mark D. Scherz

Supplementary material 4 Table S2 Data type docx file Explanation note Pairwise uncorrected distances between the 27 *Stumpffia* species used in the 16S phylogeny. https://binary.pensoft.net/file/691976This dataset is made available under the Open Database License (http://opendatacommons.org/licenses/odbl/1.0/). The Open Database License (ODbL) is a license agreement intended to allow users to freely share, modify, and use this Dataset while maintaining this same freedom for others, provided that the original source and author(s) are credited.Katherine E. Mullin, Manoa G. Rakotomanga, Jeff Dawson, Frank Glaw, Andolalao Rakotoarison, Pablo Orozco-terWengel, Mark D. Scherz
