# Supplementary material for: ﻿An unexpected new red-bellied Stumpffia (Microhylidae) from forest fragments in central Madagascar highlights remaining cryptic diversity
Source: Zookeys. 2022 Jun 6;1104:1–28. doi: 10.3897/zookeys.1104.82396 (PMC9848859; doi:10.3897/zookeys.1104.82396)
Supplement: Supplementary material 1 — Figure S1 [file zookeys-1104-001-s001.docx]

Supplementary Information


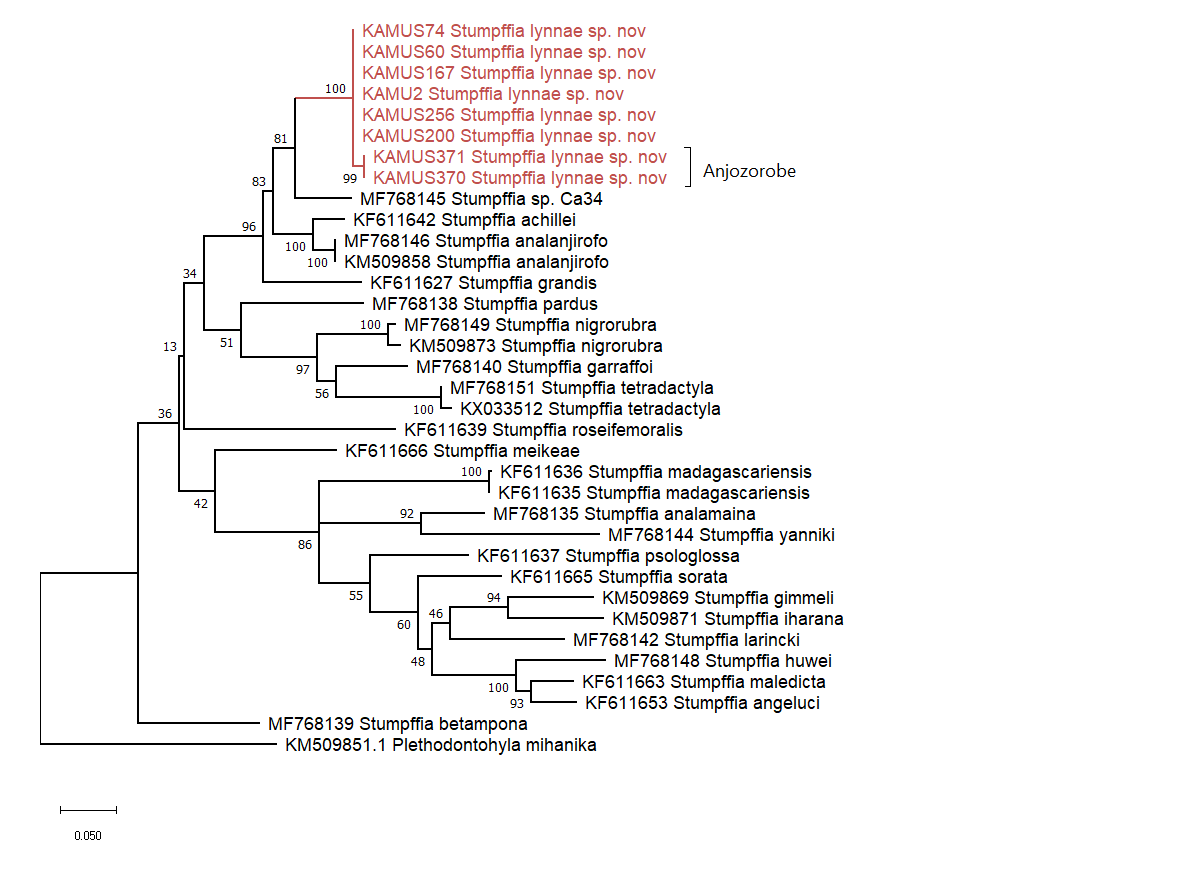


Figure S1. Maximum Likelihood phylogeny of selected Stumpffia species using a 624 bp region of the COI mitochondrial marker, using the HKY+G+I model with 1000 bootstrap replicates.
