# Supplementary material for: ﻿An unexpected new red-bellied Stumpffia (Microhylidae) from forest fragments in central Madagascar highlights remaining cryptic diversity
Source: Zookeys. 2022 Jun 6;1104:1–28. doi: 10.3897/zookeys.1104.82396 (PMC9848859; doi:10.3897/zookeys.1104.82396)

Supplementary Information

Figure S2. Sections of the 16S rRNA gene alignment, showing the differences of *Stumpffia lynnae* **sp. nov**. in comparison to 27 other *Stumpffia* species from across the *Stumpffia* phylogeny.


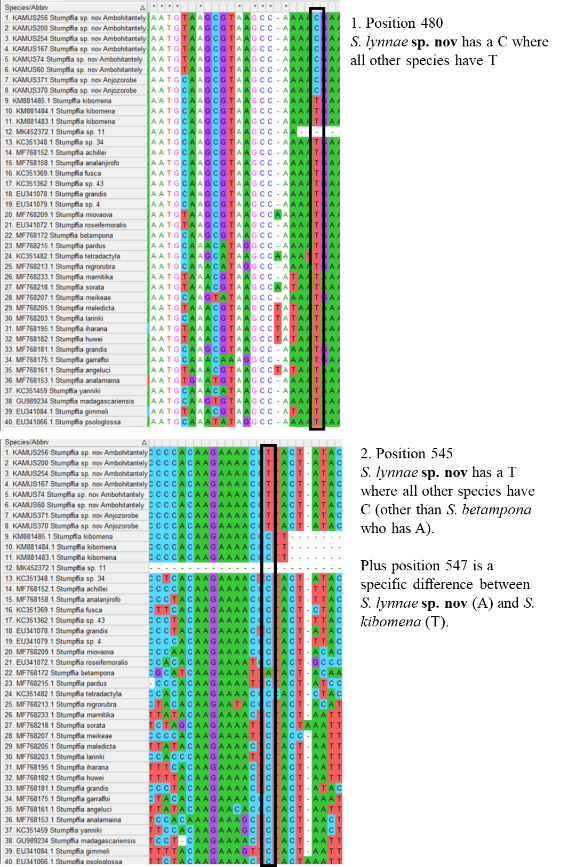


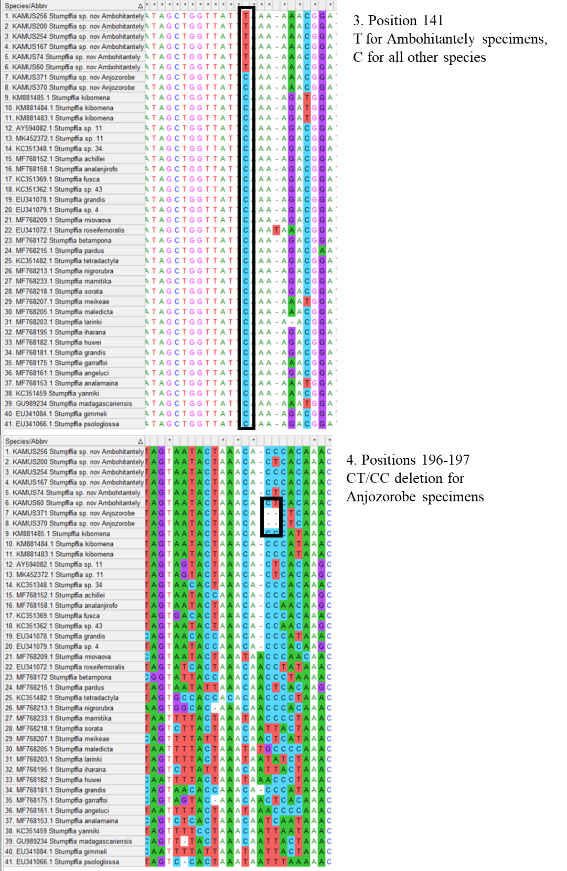

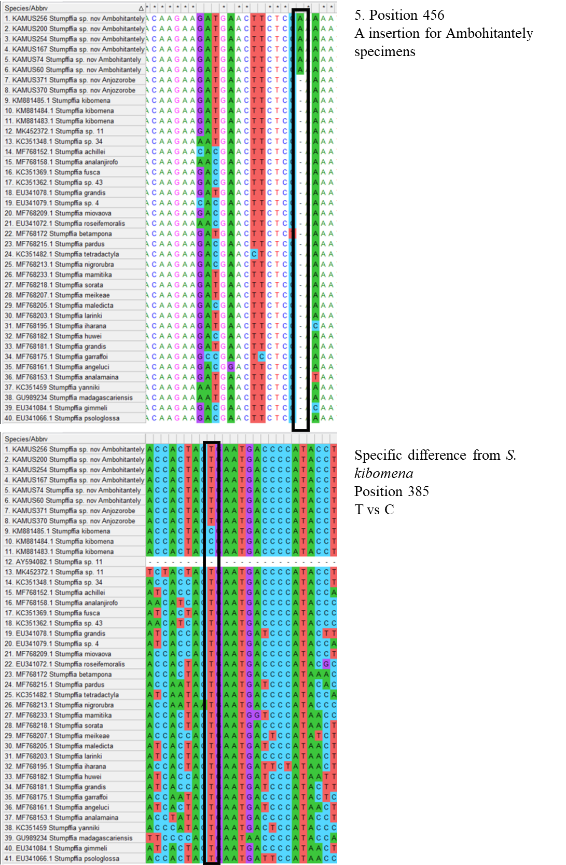

Supplement: Supplementary material 2 — Figure S2 [file zookeys-1104-001-s002.docx]
