# Supplementary material for: ﻿An unexpected new red-bellied Stumpffia (Microhylidae) from forest fragments in central Madagascar highlights remaining cryptic diversity
Source: Zookeys. 2022 Jun 6;1104:1–28. doi: 10.3897/zookeys.1104.82396 (PMC9848859; doi:10.3897/zookeys.1104.82396)
Supplement: Supplementary material 3 — Table S1 [file zookeys-1104-001-s003.docx]

Supplementary Information

Table S1. GenBank accession numbers

| **Specimen** | **5’ 16S rRNA** | **3’ 16S rRNA** | **COI** | **RAG1** |
| --- | --- | --- | --- | --- |
| KAMUS60 | ON314863 | N/A | ON313699 | ON323575 |
| KAMUS74 | ON314864 | ON332822 | ON313700 | N/A |
| KAMUS167 | ON314865 | ON332823 | ON313701 | ON323576 |
| KAMUS200 | ON314866 | ON332824 | ON313702 | ON323577 |
| ZSM 1/2022 holotype (KAMU2) | ON314867 | ON332825 | ON313703 | ON323578 |
| KAMUS256 | ON314868 | ON332826 | ON313704 | ON323579 |
| KAMUS370 | ON314869 | ON332827 | ON313705 | ON323580 |
| KAMUS371 | ON314870 | ON332828 | ON313706 | ON323581 |
