# Supplementary material for: ﻿An unexpected new red-bellied Stumpffia (Microhylidae) from forest fragments in central Madagascar highlights remaining cryptic diversity
Source: Zookeys. 2022 Jun 6;1104:1–28. doi: 10.3897/zookeys.1104.82396 (PMC9848859; doi:10.3897/zookeys.1104.82396)
Supplement: Supplementary material 4 — Table S2 [file zookeys-1104-001-s004.docx]

Supplementary Information

Table S2. Pairwise uncorrected distances between the 28 Stumpffia species (including S. lynnae) used in the 16S phylogeny.

|  |  | **1** | **2** | **3** | **4** | **5** | **6** | **7** | **8** | **9** | **10** | **11** | **12** | **13** | **14** | **15** | **16** | **17** | **18** | **19** | **20** | **21** | **22** |
| --- | --- | --- | --- | --- | --- | --- | --- | --- | --- | --- | --- | --- | --- | --- | --- | --- | --- | --- | --- | --- | --- | --- | --- |
| **1** | *achillei* |  |  |  |  |  |  |  |  |  |  |  |  |  |  |  |  |  |  |  |  |  |  |
| **2** | *analamaina* | 19.9 | 0.0 |  |  |  |  |  |  |  |  |  |  |  |  |  |  |  |  |  |  |  |  |
| **3** | *analanjirofo* | 3.8 | 18.3 |  |  |  |  |  |  |  |  |  |  |  |  |  |  |  |  |  |  |  |  |
| **4** | *angeluci* | 18.7 | 15.3 | 17.9 |  |  |  |  |  |  |  |  |  |  |  |  |  |  |  |  |  |  |  |
| **5** | *betampona* | 17.0 | 19.5 | 16.5 | 19.6 |  |  |  |  |  |  |  |  |  |  |  |  |  |  |  |  |  |  |
| **6** | *fusca* | 7.7 | 18.6 | 7.3 | 17.8 | 15.2 |  |  |  |  |  |  |  |  |  |  |  |  |  |  |  |  |  |
| **7** | *garraffoi* | 15.9 | 18.0 | 15.5 | 18.1 | 16.4 | 13.4 |  |  |  |  |  |  |  |  |  |  |  |  |  |  |  |  |
| **8** | *gimmeli* | 18.4 | 12.7 | 17.9 | 10.2 | 16.8 | 18.1 | 16.9 |  |  |  |  |  |  |  |  |  |  |  |  |  |  |  |
| **9** | *grandis* | 10.0 | 19.0 | 9.8 | 19.3 | 15.5 | 9.8 | 15.8 | 17.8 |  |  |  |  |  |  |  |  |  |  |  |  |  |  |
| **10** | *huwei* | 17.5 | 14.8 | 17.1 | 4.9 | 18.1 | 16.6 | 17.9 | 9.0 | 16.8 | 0.0 |  |  |  |  |  |  |  |  |  |  |  |  |
| **11** | *iharana* | 19.5 | 13.8 | 18.6 | 11.2 | 17.6 | 18.2 | 17.3 | 9.0 | 19.0 | 10.0 |  |  |  |  |  |  |  |  |  |  |  |  |
| **12** | *kibomena1* | 7.5 | 17.0 | 8.1 | 17.9 | 14.6 | 6.8 | 14.5 | 17.0 | 8.2 | 16.7 | 16.6 |  |  |  |  |  |  |  |  |  |  |  |
| **13** | *kibomena2* | 7.5 | 17.0 | 8.1 | 17.9 | 14.6 | 6.8 | 14.5 | 17.0 | 8.2 | 16.7 | 16.6 | 0.0 |  |  |  |  |  |  |  |  |  |  |
| **14** | *kibomena3* | 7.5 | 17.0 | 8.1 | 17.9 | 14.6 | 6.8 | 14.5 | 17.0 | 8.2 | 16.7 | 16.6 | 0.0 | 0.0 |  |  |  |  |  |  |  |  |  |
| **15** | *larinki* | 18.2 | 14.0 | 17.8 | 10.8 | 17.9 | 15.4 | 17.6 | 9.9 | 17.6 | 10.5 | 10.6 | 14.6 | 14.6 | 14.6 |  |  |  |  |  |  |  |  |
| **16** | *madagascariensis* | 18.7 | 15.1 | 17.7 | 16.6 | 19.1 | 19.5 | 19.4 | 13.9 | 19.0 | 15.7 | 14.9 | 17.5 | 17.5 | 17.5 | 13.0 |  |  |  |  |  |  |  |
| **17** | *maledicta* | 18.5 | 14.5 | 18.0 | 3.2 | 19.4 | 17.7 | 18.4 | 9.6 | 19.0 | 5.1 | 11.2 | 17.4 | 17.4 | 17.4 | 9.7 | 16.3 |  |  |  |  |  |  |
| **18** | *mamitika* | 18.6 | 14.6 | 18.1 | 5.4 | 18.3 | 17.9 | 18.8 | 10.7 | 18.1 | 5.9 | 11.5 | 17.0 | 17.0 | 17.0 | 10.9 | 15.6 | 6.2 |  |  |  |  |  |
| **19** | *meikeae* | 16.7 | 15.4 | 16.3 | 16.3 | 14.8 | 15.8 | 15.5 | 13.5 | 16.8 | 14.9 | 13.7 | 13.4 | 13.4 | 13.4 | 13.2 | 13.7 | 15.0 | 15.8 |  |  |  |  |
| **20** | *miovaova* | 12.3 | 17.5 | 11.6 | 17.7 | 12.8 | 10.8 | 15.2 | 16.2 | 10.6 | 16.2 | 17.2 | 9.5 | 9.5 | 9.5 | 16.9 | 17.6 | 17.8 | 17.3 | 14.4 |  |  |  |
| **21** | *nigrorubra* | 17.4 | 18.6 | 16.4 | 16.9 | 16.0 | 13.4 | 9.5 | 18.0 | 16.1 | 16.8 | 16.2 | 14.5 | 14.5 | 14.5 | 18.1 | 20.6 | 18.1 | 16.8 | 16.3 | 13.0 |  |  |
| **22** | *pardus* | 14.7 | 19.2 | 13.9 | 16.3 | 15.1 | 12.0 | 13.7 | 16.4 | 14.0 | 15.8 | 15.8 | 12.3 | 12.3 | 12.3 | 15.6 | 19.4 | 16.5 | 16.4 | 14.3 | 12.4 | 13.3 |  |
| **23** | *psologlossa* | 18.4 | 14.1 | 17.5 | 12.1 | 17.2 | 16.4 | 16.0 | 10.9 | 18.3 | 12.2 | 10.8 | 16.9 | 16.9 | 16.9 | 11.0 | 12.7 | 12.1 | 11.7 | 13.4 | 15.6 | 16.4 | 15.8 |
| **24** | *roseifemoralis* | 16.7 | 18.7 | 15.5 | 16.8 | 15.0 | 14.6 | 15.4 | 16.7 | 15.8 | 16.8 | 16.0 | 14.1 | 14.1 | 14.1 | 14.6 | 17.2 | 17.2 | 16.6 | 15.2 | 12.9 | 14.5 | 13.8 |
| **25** | *sorata* | 19.3 | 13.1 | 18.2 | 10.0 | 15.9 | 17.9 | 17.3 | 7.2 | 18.4 | 9.7 | 9.2 | 15.7 | 15.7 | 15.7 | 8.8 | 14.2 | 9.2 | 9.9 | 13.5 | 16.1 | 16.9 | 15.6 |

|  |  | **1** | **2** | **3** | **4** | **5** | **6** | **7** | **8** | **9** | **10** | **11** | **12** | **13** | **14** | **15** | **16** | **17** | **18** | **19** | **20** | **21** | **22** |
| --- | --- | --- | --- | --- | --- | --- | --- | --- | --- | --- | --- | --- | --- | --- | --- | --- | --- | --- | --- | --- | --- | --- | --- |
| **26** | sp. 11_1 | 9.9 | 15.6 | 9.1 | 17.6 | 15.2 | 8.0 | 11.3 | 15.1 | 10.0 | 16.4 | 13.7 | **4.9** | **4.9** | **4.9** | 15.2 | 16.1 | 17.8 | 17.3 | 13.1 | 12.2 | 11.6 | 10.4 |
| **27** | sp. 11_2 | 9.2 | 16.0 | 9.1 | 17.7 | 12.9 | 6.9 | 13.4 | 15.9 | 9.3 | 16.6 | 16.0 | **4.1** | **4.1** | **4.1** | 14.4 | 16.6 | 17.8 | 17.4 | 14.0 | 11.2 | 14.0 | 12.1 |
| **28** | sp. 34 | 7.9 | 17.0 | 7.7 | 17.6 | 14.7 | 7.2 | 15.5 | 17.0 | 9.1 | 16.0 | 18.6 | **6.0** | **6.0** | **6.0** | 15.3 | 17.0 | 16.8 | 17.2 | 15.6 | 10.9 | 15.4 | 13.7 |
| **29** | ***sp. nov.*** *lynnae Amb* | 8.9 | 17.4 | 8.3 | 16.8 | 15.8 | 6.8 | 14.0 | 16.3 | 8.1 | 16.2 | 16.1 | **3.8** | **3.8** | **3.8** | 14.8 | 17.9 | 16.4 | 16.3 | 14.6 | 9.9 | 14.4 | 11.9 |
| **30** | ***sp. nov.*** *lynnae Amb* | 9.0 | 17.6 | 8.5 | 17.0 | 16.0 | 7.0 | 13.8 | 16.2 | 8.2 | 16.4 | 15.9 | **3.9** | **3.9** | **3.9** | 14.8 | 17.7 | 16.6 | 16.4 | 14.5 | 10.1 | 14.6 | 11.7 |
| **31** | ***sp. nov.*** *lynnae Amb* | 8.9 | 17.4 | 8.3 | 16.8 | 15.8 | 6.8 | 14.0 | 16.3 | 8.1 | 16.2 | 16.1 | **3.8** | **3.8** | **3.8** | 14.8 | 17.9 | 16.4 | 16.3 | 14.6 | 9.9 | 14.4 | 11.9 |
| **32** | ***sp. nov.*** *lynnae Amb* | 8.9 | 17.4 | 8.3 | 16.8 | 15.8 | 6.8 | 14.0 | 16.3 | 8.1 | 16.2 | 16.1 | **3.8** | **3.8** | **3.8** | 14.8 | 17.9 | 16.4 | 16.3 | 14.6 | 9.9 | 14.4 | 11.9 |
| **33** | ***sp. nov.*** *lynnae Amb* | 9.0 | 17.6 | 8.5 | 17.0 | 16.0 | 7.0 | 13.8 | 16.2 | 8.2 | 16.4 | 15.9 | **3.9** | **3.9** | **3.9** | 14.8 | 17.7 | 16.6 | 16.4 | 14.5 | 10.1 | 14.6 | 11.7 |
| **34** | ***sp. nov.*** *lynnae Amb* | 9.0 | 17.6 | 8.5 | 17.0 | 16.0 | 7.0 | 13.8 | 16.2 | 8.2 | 16.4 | 15.9 | **3.9** | **3.9** | **3.9** | 14.8 | 17.7 | 16.6 | 16.4 | 14.5 | 10.1 | 14.6 | 11.7 |
| **35** | ***sp. nov.*** *lynnae Anj* | 8.9 | 17.7 | 8.4 | 16.9 | 15.2 | 7.0 | 13.8 | 16.1 | 8.2 | 16.3 | 16.0 | **3.6** | **3.6** | **3.6** | 14.6 | 17.6 | 16.4 | 16.8 | 14.5 | 10.5 | 14.1 | 11.9 |
| **36** | ***sp. nov.*** *lynnae Anj* | 8.9 | 17.7 | 8.4 | 16.9 | 15.2 | 7.0 | 13.8 | 16.0 | 8.2 | 16.2 | 15.9 | **3.6** | **3.6** | **3.6** | 14.6 | 17.5 | 16.4 | 16.8 | 14.5 | 10.5 | 14.1 | 11.9 |
| **37** | *tetradactyla* | 15.4 | 20.1 | 16.1 | 18.5 | 17.6 | 12.8 | 15.5 | 18.8 | 15.5 | 17.6 | 19.9 | 13.9 | 13.9 | 13.9 | 16.8 | 19.8 | 18.8 | 18.3 | 17.2 | 14.8 | 14.1 | 13.8 |
| **38** | *yanniki* | 20.3 | 11.8 | 18.4 | 16.2 | 18.7 | 18.7 | 17.3 | 14.0 | 19.5 | 15.9 | 14.7 | 17.0 | 17.0 | 17.0 | 15.1 | 14.8 | 16.1 | 15.2 | 15.2 | 19.2 | 17.7 | 16.1 |

|  |  | **23** | **24** | **25** | **26** | **27** | **28** | **29** | **30** | **31** | **32** | **33** | **34** | **35** | **36** | **37** | **38** |
| --- | --- | --- | --- | --- | --- | --- | --- | --- | --- | --- | --- | --- | --- | --- | --- | --- | --- |
| **23** | *psologlossa* |  |  |  |  |  |  |  |  |  |  |  |  |  |  |  |  |
| **24** | *roseifemoralis* | 15.5 |  |  |  |  |  |  |  |  |  |  |  |  |  |  |  |
| **25** | *sorata* | 10.8 | 15.8 |  |  |  |  |  |  |  |  |  |  |  |  |  |  |
| **26** | sp. 11_1 | 14.3 | 14.1 | 15.2 |  |  |  |  |  |  |  |  |  |  |  |  |  |
| **27** | sp. 11_2 | 16.3 | 14.7 | 15.8 | 1.8 |  |  |  |  |  |  |  |  |  |  |  |  |
| **28** | sp. 34 | 15.7 | 14.0 | 16.5 | 8.0 | 7.2 |  |  |  |  |  |  |  |  |  |  |  |
| **29** | ***sp. nov.*** *lynnae Amb* | 16.7 | 13.5 | 16.0 | **5.0** | **4.5** | **7.0** |  |  |  |  |  |  |  |  |  |  |
| **30** | ***sp. nov.*** *lynnae Amb* | 16.5 | 13.7 | 15.8 | **4.7** | **4.3** | **7.2** | 0.2 |  |  |  |  |  |  |  |  |  |
| **31** | ***sp. nov.*** *lynnae Amb* | 16.7 | 13.5 | 16.0 | **5.0** | **4.5** | **7.0** | 0.0 | 0.2 |  |  |  |  |  |  |  |  |
| **32** | ***sp. nov.*** *lynnae Amb* | 16.7 | 13.5 | 16.0 | **5.0** | **4.5** | **7.0** | 0.0 | 0.2 | 0.0 |  |  |  |  |  |  |  |
| **32** | ***sp. nov.*** *lynnae Amb* | 16.5 | 13.7 | 15.8 | **4.7** | **4.3** | **7.2** | 0.2 | 0.0 | 0.2 | 0.2 |  |  |  |  |  |  |
| **34** | ***sp. nov.*** *lynnae Amb* | 16.5 | 13.7 | 15.8 | **4.7** | **4.3** | **7.2** | 0.2 | 0.0 | 0.2 | 0.2 | 0.0 |  |  |  |  |  |
| **35** | ***sp. nov.*** *lynnae Anj* | 16.6 | 13.8 | 15.8 | **4.4** | **4.1** | **7.0** | **1.3** | **1.3** | **1.3** | **1.3** | **1.3** | **1.3** |  |  |  |  |
| **36** | ***sp. nov.*** *lynnae Anj* | 16.5 | 13.7 | 15.8 | **4.4** | **4.1** | **7.0** | **1.3** | **1.3** | **1.3** | **1.3** | **1.3** | **1.3** | 0.0 |  |  |  |
| **37** | *tetradactyla* | 18.1 | 14.1 | 17.8 | 12.7 | 13.4 | 15.1 | 14.4 | 14.6 | 14.4 | 14.4 | 14.6 | 14.6 | 14.5 | 14.5 |  |  |
| **38** | *yanniki* | 13.8 | 17.7 | 13.8 | 14.2 | 15.7 | 17.0 | 17.7 | 17.5 | 17.7 | 17.7 | 17.5 | 17.5 | 17.2 | 17.2 | 19.3 |  |
